# Ensuring sustained ACT production and reliable artemisinin supply

**DOI:** 10.1186/1475-2875-6-125

**Published:** 2007-09-15

**Authors:** Jean-Marie Kindermans, Jacques Pilloy, Piero Olliaro, Melba Gomes

**Affiliations:** 1 AEDES Foundation, 34, rue Joseph II, 1000, Brussels, Belgium; 2 OTECI, 10, rue du Havre, 75009, Paris, France; 3 UNICEF/UNPD/WB/WHO Special Programme for Research and Training in Tropical Diseases (TDR), 2, avenue Appia, CH1211 Geneva 27, Switzerland; 4 Centre for Tropical Medicine and Vaccinology, University of Oxford, Oxford, UK

## Abstract

**Introduction:**

This paper reviews recent trends in the production, supply and price of the active ingredients as well as finished ACT products. Production and cost data provided in this paper are based on an ongoing project (Artepal). Stability data are derived from a development project on rectal artesunate.

**Discussion:**

The artemisinin raw material and its derivatives appear to be very stable compared to the finished products. Supply of artemisinin changed in May 2004 when the Global Fund shifted financial support to qualified countries from chloroquine or sulphadoxine-pyrimethamine to an ACT for treatment of malaria. First, there was a sudden shortage of the starting material, and short term scarcity led to a steep rise in API price: it increased dramatically in 2004, from $350 per kg to more than $1000. Second, there was a parallel increase in the number of companies extracting artemisinin from 10 to 80 between 2003 and 2005 in China, and from 3 to 20 in Vietnam. Commercial cultivation began also in East Africa and Madagascar.

A steady and predictable demand for the crop can eliminate such wide fluctuations and indirectly contribute to price stability of the herb, the API and ACT. With appropriate mechanisms to reduce those fluctuations, the cost of artemisinin might decrease sustainably to US$ 250–300 per kg.

**Conclusion:**

Today the global health community is facing the risk of another cyclical swing with lower demand feeding into reduced planting of *A. annua *and, thereafter, a new shortage of the raw material and higher API prices. International donors, the largest purchasers for ACTs could better coordinate their activities, in order to guarantee purchase of ACTs and consequently of API with manufacturers. In parallel, the base of quality producers of APIs and finished ACT products needs to be broadened.

While the ACT programme is still in its early stages, the consequences of another wave of artemisinin and ACT shortages would permanently discredit it and impede any progress in rolling malaria back.

## Introduction

Artemisinin compounds, derived from *Artemisia annua*, also known as sweet wormwood, have become the cornerstone of malaria case-management whether used orally for the treatment of uncomplicated malaria episodes or in parenteral or rectal administration for severe malaria. The World Health Organization (WHO) now recommends treating uncomplicated malaria with artemisinin-based combinations therapies (ACTs) [1]. This new policy was issued to protect the artemisinins and the partner drug from resistance in the wake of reduced efficacy of the well-known once-first-line, antimalarials (chloroquine, and sulphadoxine-pyrimethamine) due to resistance. ACTs are much more expensive and have a shorter shelf-life than the traditional antimalarials. They consequently require substantially improved post-production supply-chain support in malaria endemic countries.

WHO's guidance on ACTs first issued in 2001 has just been re-stated with evidence justifying the recommendations [2], but the uptake of ACT continues to be low despite increased donor funding.

This paper reviews recent trends in the production, supply and price of the active ingredients as well as finished ACT products, and identifies factors that need to be taken into account in stabilizing the artemisinin market and lowering the price of ACTs to achieve wider use of these important medicines.

Production and cost data provided in this paper are based on field visits, interviews, and indirect verification undertaken in the course of an ongoing project (Artepal) that seeks to facilitate transfer of ACT technology to Africa and Asia, keep producers and potential clients informed about global artemisinin production and consumption, and provide public information regarding difficulties producers are facing (see website http://www.artepal.org). Stability data are derived from an ongoing development project on rectal artesunate.

## Discussion

### From *Artemisia annua *to ACT

#### (i) *A.annua *cultivation and production of artemisinin

The world's production of *Artemisia annua *is, at present, dominated by cultivation in East Asia (mainly China and Vietnam) with recent additions of crop in East and Southern Africa, including Madagascar.

Harvesting of *A. annua *takes place before flowering when the artemisinin yield is highest, and is completed within a month. Extraction takes place during four to six months after the harvest which occurs around six months after planting. Thus a period of 12 months from the time of planting of *A. annua *is required to first produce the bulk artemisinin starting material and eventually the finished product.

The average yield of *A. annua *varies between 1.5 to 2 tons of dried matter per hectare and the average content of artemisinin varies with the seed quality, climate, altitude, soil, farmers know-know, and planting density. In general, it lies between 0.5–1.2%, while extraction and purification yield are in the range of 50–80%. These parameters may change with time as cultivation methods further improve [3], but their present value lead to producing from 6 to 14 kgs of artemisinin raw material per hectare.

In order to bypass the bottleneck associated with plant production, investments are being made to obtain substitutes sharing the same trioxane active moiety of artemisinin by total synthesis; one such molecule is currently in clinical development. However, plant production of *A. annua *will remain indispensable to the ACT programme for the foreseeable future.

#### (ii) Production and stability of the active pharmaceutical ingredient (API)

The artemisinin "raw" or "starting material" extracted from the dried plant is transformed into artemisinin based APIs produced through further hemi-synthesis of artemisinin. Transformation of artemisinin into any one of these APIs – dihydroartemisinin (DHA), artesunate (AS), artemether (AM) or arteether (AE) – requires industrial facilities and further manufacturing.

The active pharmaceutical ingredient proves to be stable for all the APIs for which we have data. Table 1 shows stability data for most of the artemisinin derivatives – artemisinin, dihydroartemisinin and artesunate, expressed in percentage of API content. Data were provided by Abbott/Knoll for artesunate and IPCA for artemisinin and dihydroartemisinin, then consolidated by WHO/TDR (Tropical Disease Research).

**Table 1:** Stability data of artemisinin derivatives, in percentage of API content.

**Product and conditions**	**Specification**	**12 months**	**24 months**	**36 months**	**48 months**	**60 months**
**Artemisinin 30°C RH 65%**	99,0%	99,6%	100,0%	99,6%	not tested	not tested
**Dihydroartemisinin 30°C, RH 65%**	98%–102%	99,2%	99,4%	99,8%	99,7%	not tested
**Artesunate 25°C RH 60%**	98–102%	99,5%	100,5%	99,1%	99,6%	99,8%
**Artesunate 30°C RH 70%**	98–102%	101,3%	101,4%	100,8%	not tested	not tested

The bulk drug substance is seen to be very stable at temperatures of 30°C and 65% relative humidity (RH), conditions required for stability testing for climatic zones III (hot and dry) and IV (hot and humid). Data are available for three years for API stored at 30°C and RH of 70% and for five years for API of artesunate stored at 25°C and RH 60%. Data are not available for artemether.

No term tests of stability longer than 60 months have so far been conducted for these APIs.

#### (iii) Production and stability of the final finished product – ACT

ACTs are currently provided either as two independently-formulated and co-blistered products (the artemisinin component and the companion drug), or as fixed-dose co-formulations. The development of the final pharmaceutical dosage form in ACTs is complicated by the fact that there are two active ingredients, i.e. two APIs each requiring independent production and final formulation as a fixed-dose combination with compatible excipient (s).

The shelf-life specified by regulatory authorities for the final ACT dosage forms is much shorter than that of the API. No stringent regulatory agency has approved a shelf-life that exceed 24 months for any oral formulations of artemisinin-based combinations.

### Factors affecting ACT availability

#### (i) Supply of artemisinin

**Figure 1:**
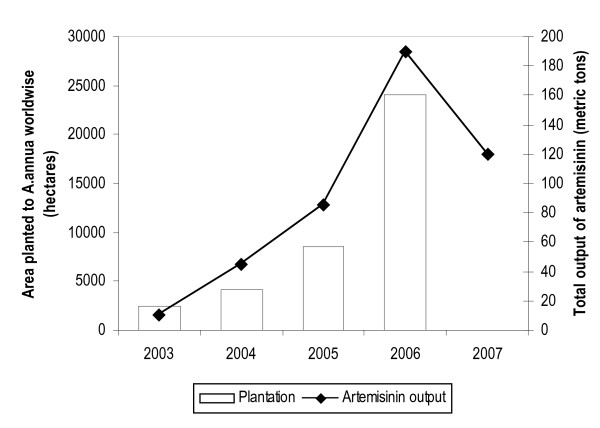
areas planted to *A.annua *and output of artemisinin from 2003 to 2006, with 2007 projections.

Initially, artemisinin-based products were manufactured and used almost exclusively in China and Vietnam. During this period, it was not difficult for agricultural production to meet stable domestic or regional requirements. The situation changed in May 2004 when the Global Fund for AIDS, Tuberculosis and Malaria (GFATM) shifted financial support to qualified countries from chloroquine or sulphadoxine-pyrimethamine therapy to an ACT for treatment of malaria. At the time, the WHO estimated the ACT demand to be 10 million treatment courses for 2004 and 60 million for 2005 [4]. The announcement that the GFATM would shift funding support to ACTs by $200 million in the wake of an intense exchange between the GFATM and proponents of combination therapy [5], had two consequences, not fully anticipated at the time, which are illustrated on Figure 1.

First, there was a sudden shortage of the starting material, and short term scarcity led to a steep rise in API price about which more is said below. At the time the GFATM resolution was made, farmers had already made decisions regarding the total area they would plant to different crops, including *A. annua*, on the basis of past market prices for artemisinin versus other commercial crops. The emerging shortage was widely reported and a likely increase in production forecast [6].

Second, propelled by the prospect of increased prices, the total acreage devoted to plantations of *A. annua *indeed increased substantially in China and Vietnam in the December 2005 planting season, the farmers now reacting to the 2005 reality and prospect of higher prices in 2006. There was a parallel increase in the number of companies extracting artemisinin from 10 to 80 between 2003 and 2005 in China, and from 3 to 20 during the same period in Vietnam. Commercial cultivation began in East Africa and Madagascar.

Afterwards, with 2006 reductions in price of the API and uncertainty about future demand for the artemisinin API, factors discussed in sections that follow, the rate of new planting has begun to fall and a fall is predicted for 2007.

Figure 1 provides data from the Artepal project on estimated area planted (in hectares with log scale) to *A.annua *from 2003 to 2006, and the output of artemisinin (in metric tons) during the period 2003 to 2006, with 2007 projections.

#### (ii) The market for *A. annua*, artemisinin and the packaged product

The demand for *A. annua *derives from the demand for the finished formulated product by customers and purchasers (health services and individuals in malaria-endemic countries, international organizations). The price of the product plays a role at all levels. The supplementary cost of ACTs compared with chloroquine has meant that many health services in these countries depend on donor support for ACT procurement. Like malaria patients and health services, donors themselves have an interest in seeing the prices of ACT as low as possible to ensure that available budgets can reach the largest number possible of malaria patients.

Any increase in the price of artemisinin – in turn depending on the price of *A. annua *– contributes to the ultimate cost of ACT and is, therefore, of major concern to policy and practice. The API price of the artemisinin component typically accounts for 20 to 40 per cent of the final price of the finished ACT product, depending on the companion drug in the ACT [7].

The supply of *A. annua *plays an equally important role. For a given level of demand by product manufacturers, a greater supply of the herb contributes to lowering its price while a temporary shortage can drive up its price and those of artemisinin final product. Not unlike other non-regulated commercial crops, demand fluctuations can set off a cycle of supply adjustments and price volatility as producers adjust the area planted on the basis of their price expectations. A year of abundance of the raw material can quickly turn into a year of relative scarcity as happened recently. A steady and predictable demand for the crop can eliminate such wide fluctuations and indirectly contribute to price stability of the herb, the API and ACT. Other factors such as the increased yield of *A. annua *due to agronomical improvements or the efficiency of the API extraction process can, in favourable circumstances, permanently lower the price of both the crop and the API.

#### (iii) Demand for ACTs

How much is at stake? The upper limit to the demand for ACT treatment would be given by the simple number of malaria sufferers (times the appropriate dose in each case), a point that might be reached if ACT were to be distributed free of charge and at the right moment. Current global and country estimates of the upper limit ("the need", to the medical profession) vary greatly and are not particularly reliable given generally inaccurate country reporting and a widespread practice of treating many fevers (rather than only parasitologically-confirmed malaria) with antimalarials. Available estimates include 2.84 billion fever episodes per annum in Africa [8] and a range of estimates of *falciparum *clinical malaria cases from 226 million [9] to 365 (215–374 million) [10]. Using a consumption method with old antimalarials, the projected lower and upper number of adult treatments was estimated at 113–314.5 million equivalent adult treatments for the continent [11].

**Figure 2:**
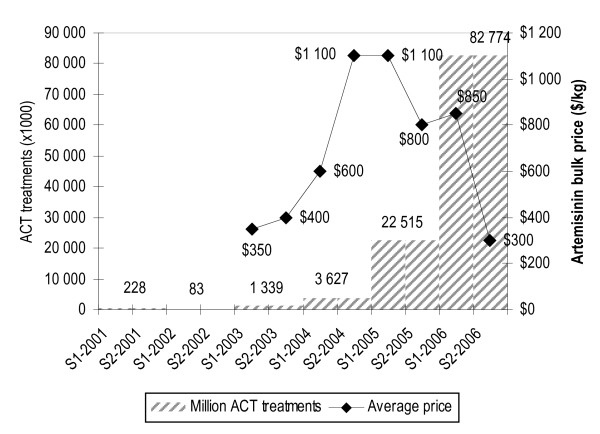
recorded orders of ACTs by the public sector (courtesy of Marise Dugue, WHO/GMP), and market prices of bulk artemisinin for small orders (Artepal project).

The above figures can be compared to the number of actual recorded orders for ACT treatments by the public sector mostly by the Global Fund (Figure 2), showing that the continent's current consumption of ACT falls well short of the medical need.

#### (iv) Trends in artemisinin prices

The prices of artemisinin API during the last three years increased dramatically in 2004, from $350 per kg to more than $1,000. This trend was confirmed by other sources [6].

Such fluctuations are striking but the pattern is not surprising. The peak of prices occurred in 2004 (stimulated by official support given to ACT by WHO), before additional output from extra planting of 2005 and 2006 could materialize. The rapid drop in prices of 2006 appears to reflect the major increase in production in 2006, a year that saw orders stay below 100 million treatments, contrary to earlier expectations and predictions. Prices have decreased to US$ 250 per kg at the end of 2006 and first half of 2007 (with spot prices under US$ 200). Hectarage planted in late 2006–2007 has likely reverted to its previous low base. In the presence of mechanisms that reduce wide demand fluctuations – one of which might be a system of guaranteed purchases – the cost of artemisinin might decrease sustainably to US$ 250–300 per kg.

## Conclusion

High prices and temporary artemisinin shortages such as those experienced in 2004/2005 are a serious threat to the ACT programme. High and unstable API prices have a negative impact on ACT affordability and have been a psychological factor undermining confidence in ACT availability and affordability in the future.

As this article is going to press (third quarter of 2007), there is a glut of artemisinin and a number of artemisinin producers (in China, for instance) have announced plans to reduce or discontinue production. The global health community is facing the risk of another cyclical swing with lower demand feeding into reduced planting of *A. annua *and, thereafter, a new shortage of the raw material and higher API prices. The market for artemisinin is not mature and in the absence of demand-stabilizing factors the periodic fluctuations are likely to undermine faith in the direction of the malaria treatment advocated by WHO in spite of increased funding for ACT. For this to be avoided, several things need to happen.

First, market demand needs to be stabilized. ACT purchases are special in the sense of being dominated by a relatively small number of donor organizations. The majority of individual country orders are made via submissions to the Global Fund (sometimes via WHO), as well as through procurement by organizations such as the President Malaria Initiative or the World Bank. These organisations – the largest purchasers for ACTs by far – could better coordinate their activities, in order to guarantee purchase of ACTs and consequently of API to manufacturers, thus mitigating market instability and obtaining the lowest possible prices. For now, the donor community purchasing ACTs does not send coordinated or consistent messages to producers, either those of the API or of the finished product. By default, the market is determined largely by the main manufacturers of finished products via their forward contracts with artemisinin producers designed to secure the raw material for their own products. An opportunity to bring about more stable prices of the raw material for everybody and lower the prices of artemisinin API is lost.

Such a coordination would include consolidation of global orders and better frameworks regarding contract agreements (as happens, for instance, in vaccine procurement). The aim should be steady (and possibly guaranteed) prices for bulk raw material which must also be fair to the producers and farmers.

In the meanwhile, the relatively long stability of the APIs could also be used to moderate short-term API price fluctuations by developing a mechanism for guaranteed purchases.

Second, and in parallel, the base of quality producers of APIs and finished ACT products needs to be broadened. This is key to improving the availability of GMP production and increasing competition among the API and ACT producers (and thus minimizing unnecessary price increases). Subsidizing costs of meeting the international criteria and gaining WHO certification might be considered, for instance, for those companies unable to invest in costly bio-equivalence studies, generally necessary for prequalification and access to public sector bidding.

While these are the two main requirements emerging from this analysis, it is clear that in order for ACTs to be successfully implemented, other conditions should also be met. ACTs are an expensive product with a relatively short shelf life, and need to be ordered and used in time. Shortening the lag from production to use through a more effective supply and distribution chain support in malaria endemic countries is an essential long-term policy objective.

The comparatively short shelf life of the final product is also a critical consideration as manufacturers risk overproduction.

Progress on the above issues is a matter of some urgency requiring concrete public action. While the ACT programme is still in its early stages, the consequences of another wave of artemisinin and ACT shortages would permanently discredit it and impede any progress in rolling malaria back.

## Authors' contributions

JMK and JP conceived the article. JP collected data on production and prices with support of JMK, both from the Artepal project. MG collected stability data. JMK, PO and MG wrote the manuscript, with support from JP.

The final version of the manuscript was seen and approved by all authors.
